# Emotion regulation skills as a mediator of STEM teachers’ stress, well-being, and burnout

**DOI:** 10.1038/s41598-024-63228-z

**Published:** 2024-07-06

**Authors:** Moran Farhi, Orly Rubinsten

**Affiliations:** 1 The MOFET Institute, Tel Aviv, Israel; 2 David Yellin College, Jerusalem, Israel; 3 Department of Learning Disabilities, Edmond J. Safra Brain Research Center for the Study of Learning Disabilities, University of Haifa, Haifa, Israel

**Keywords:** Emotion regulation, Well-being, Burnout, Stress, STEM teachers, Human behaviour, Quality of life

## Abstract

The teaching profession highly stressful, and teachers are often faced with challenging situations. This is particularly the case in STEM (science, technology, engineering, and math) education, which is a uniquely demanding and challenging field. This study examined the role of emotional regulation (ER) skills in STEM teachers’ stress, well-being, and burnout. The sample included 165 STEM teachers in middle and high schools who completed standard online questionnaires on ER, stress, well-being, and burnout. They were also asked to comment on three videos depicting authentic mathematical and pedagogical situations. The results indicated that contrary to popular belief, seniority was not linked with levels of stress, difficulties in ER, lower levels of well-being, or higher levels of burnout. A structural equation model and bootstrapping analysis showed teachers’ levels of stress predicted their well-being, and this link between stress and well-being was mediated by teachers’ level of difficulty in ER. The study highlights the importance of STEM teachers’ well-being and suggests the need to reduce stress and burnout by providing tools for teachers to regulate their emotions in the classroom.

## Introduction

A teacher's day at school includes interactions with students, staff, parents, and management. The teacher must meet management's requirements, navigate relationships with team members, create relationships with students, and talk to parents. Given this range, interactions in the school space can become emotionally overwhelming. Studies show teachers in certain subject areas, such as STEM (science, technology, engineering, and math), may encounter more stress than others. For example, with the increased importance of technology in Western societies, students are encouraged to master STEM subjects^1^. Parents and administrators may have high expectations of STEM teachers, seeing them as responsible for student achievement^2^, and this, in turn, may lead to increased stress, burnout, and reduced well-being. There is also a shortage of teachers in STEM fields^3^, largely due to teachers leaving the field. As emotion regulation (ER) has been found helpful in reducing stress and promoting well-being, STEM education research is focusing attention on emotion and ER^4^ in attempts to ameliorate the situation.

This study investigated the emotion management of STEM teachers, that is, their ability to regulate their emotions and the relationship between their ER and feelings of stress, mental well-being, and burnout. In what follows, we first describe the emotional aspects associated with the teaching profession, focusing on stress, well-being, and burnout, and the consequences of teachers' emotional state on teacher-student relationships. How teacher characteristics affect student education is of significant interest, with research primarily focusing on teachers’ educational background^5^, seniority^6^, and salaries^7^. Such research, while plentiful, has failed to reach consensus on the causal impact of teachers’ characteristics and coping mechanisms. We therefore investigated how STEM teachers’ emotional states affect teachers’ outcomes. We tested ER skills as a central tool that STEM teachers may use to improve their relationships with students, increase their well-being, and reduce their stress and burnout.

### Teachers’ stress, well-being, and burnout

The emotional states of teachers have a central role in teaching-related outcomes^8–10^. Research has found teacher–student relationships, which are partially influenced by teachers’ emotional states, are significantly associated with children’s long-term academic achievement^11–14^ and have an impact on emotional aspects for both students^15^ and teachers^16,17^. The emotions of teachers have effects on their physical and psychological well-being, professional engagement, burnout, and turnover^11,18–21^. Previous studies have found a correlation between burnout and well-being^22,23^ and between burnout and stress^24,25^.

#### Teachers’ stress

Teacher stress is defined as the experience of negative and unpleasant emotions stemming from everyday work^26^. A recent study found teachers and school principals experience job-related stress at a rate that is twice as high as that of the general working population^27^. In the United States, 46% of K-12 teachers report experiencing high levels of daily stress^28^.Teaching is an extremely stressful profession^29^ because of high job demands, excessive workloads, student misbehavior, and high or unrealistic expectations from authorities, students, and parents^30,31^.Studies suggest stress is especially prevalent among novice teachers^32^, possibly because of their lack of experience and the rapid shift from student to teacher^33^. One study found a quarter of novice teachers are at risk for stress in the first year of their work^34^.

Recent studies found teachers' emotional state worsened with a return to in-class learning following the COVID-19 crisis^35^. Problematically, high levels of stress may cause teachers to leave the profession^36^. Elevated stress levels have been linked to a variety of negative health outcomes, including fatigue and sleep disruption^28^.

#### Teachers’ well-being

Stress has a significant impact on the overall well-being of teachers^19^. Well-being relates to life quality and satisfaction^37^ and has been explained as ‘subjective, positivity and evaluation of one’s whole life generally’^38^. A systematic review of 98 studies on teacher well-being from 2000 to 2019 found well-being influences teaching quality^39^. In another study, teachers’ well-being was associated negatively with teacher stress and burnout^11^.

### Teachers’ burnout

Burnout has been defined as a psychological syndrome that involves a prolonged response to stressors in the workplace^40^. Job burnout includes emotional exhaustion, whereby workers feel mental and physical fatigue. It is commonly observed among those who work under heavy pressure and prolonged stress, mostly in helping professions, such as the medical field or teaching^25,41,42^. Teachers commonly feel a sense of burnout^43^. The pressure they experience in their work causes physical and mental exhaustion and stems from several factors, including intense relationships with colleagues, students, and parents, as well as long working hours^44^. Teacher burnout has an effect on students as well; in a systematic review, Madigan and Kim found teacher burnout is associated with decreased student academic achievement and reduced student motivation^45^.

In this study, we investigated three dimensions of burnout: exhaustion, causing teachers to feel they are no longer able to give of themselves at a psychological level; depersonalization of students, whereby teachers develop negative cynical attitudes to their students; lack of self-fulfillment, whereby teachers evaluate themselves negatively, particularly in regard to their work with students^25^.

#### Teacher ER

These emotional states (i.e., feelings of stress, well-being, burnout) affect the way teachers evaluate or appraise a situation (i.e., cognitive appraisal). However, ER is a tool teachers may use to change their appraisals, thus reducing their stress and burnout and increasing their well-being^46,47^. ER refers to the processes by which people influence which emotions they have, when they have them, and how they experience and express them^48,49^. There are four common ER strategies. Two are maladaptive: rumination (focusing on negative aspects in a passive and repetitive way) and suppression (inhibiting immediate emotional responses)^50,51^. Two are adaptive: acceptance (accepting feelings without trying to control or judge them) and reappraisal (reinterpreting the meaning of an event or its outcome to change its emotional trajectory)^52–54^. We focused on suppression and reappraisal.

Individuals who have effective ER show a clear understanding of their emotions and the context in which they are experiencing them. They are also able to identify and prioritize their ER goals. To have effective ER skills, individuals must make a deliberate and skilled choice of ER strategy, while being mindful of their goals and adapting their approach as needed in response to changes in the situation^55^.

It is important to have a wide range of strategies and to be able to select strategies that align with the situation and personal goals. This is known as ER flexibility^56^. Emotion dysregulation is defined as a difficulty in managing emotional states^57^ and having little or no ER flexibility. Difficulties in ER can manifest as either the failure to use appropriate ER strategies or the use of inappropriate or maladaptive strategies^12^. We focused on difficulties in ER as a potential mediator between stress, well-being, and burnout.

#### Importance of adaptive ER

From an intra-personal perspective, teachers who demonstrate adaptive ER^50^ exhibit more effective pedagogical behaviors^47,58,59^ and report better psychological well-being^60,61^. On an inter-personal level, teachers may impact the ER tendencies of their students^12,62^ by modeling effective strategy use^63^, and this, in turn, may lead to enhanced academic performance among students^12,62^. Moreover, teachers' ER is positively related to teacher-student interactions^64^ and students' well-being^50^. Hence, teachers’ emotional state and their ability to regulate it may be no less important than the more widely studied education or general experience measures. In fact, the ability to efficiently regulate emotions should be considered a crucial component of teaching^29^.

Reappraisal is an effective ER strategy in the field of education^49,55,65^. Teachers who frequently regulate their emotions through avoidance or suppression are more likely to experience burnout^58,66^. Suppression, in particular, may lead to increased anxiety, strain, and emotional exhaustion in teachers^21,59,66^.

A recent intervention study suggested reappraisal strategies help teachers deal with environmental stressors and are related to decreased stress^29^. Chang and Taxer found teachers in general use a variety of strategies to regulate their emotions^67^. Teachers who typically reappraise have the least negative affective experiences in the context of student misbehavior and are less likely to suppress their in-the-moment negative emotions.

#### STEM teachers and ER

To the best of our knowledge, no one has investigated emotions and ER in STEM teachers, even though the relationship between stress, well-being, and burnout is especially relevant for these teachers. First, Western societies emphasize STEM subjects^1^, seeing them as crucial to students’ future success. This leads to pressure being placed on students^68,69^ and also on STEM teachers^70^. Teachers of STEM subjects encounter pressure from various sources, including the education system, parents, and school administration, to show high student performance^2^. Second, the pressure on teachers increases if students perform poorly. For example, Israel recently ranked 41st in mathematics and 42nd in science out of 78 countries in the Program for International Student Assessment (PISA) , and this ranking suggested the need for Israeli teachers to do better^71^. Third, STEM subjects are known to be challenging to teach^72,73^. These factors make STEM teachers an especially interesting population in which to study stress, burnout, and well-being.

### The current study

We examined the relationship between STEM teachers’ ER skills and stress, well-being, and burnout. We formulated five hypotheses:(A) Increased stress levels among STEM teachers will be linked to difficulties in ER^21,58,66^ which, in turn, will be associated with decreased well-being and increased burnout^58,59,66^.

(B) Difficulties in ER will be associated with the use of ER strategies. There will be a positive correlation with the use of suppression and a negative correlation with the use of reappraisal. Findings from Gross's lab show suppression is the most frequently used ER strategy among teachers in the classroom^74^.

(C) Seniority as a continuous variable will correlate with stress^32–34^.

(D) There will be a correlation between the two ER self-report questionnaires, Emotion Regulation Questionnaire (ERQ) and Difficulties in Emotion Regulation Scale (DERS), and participants’ responses to authentic mathematical and pedagogical situations shown in videos. Studies have demonstrated the benefits of using videos as tools for ER evaluation^75–77^. First, film clips are less likely to be seen as deceptive and can evoke strong behavioral and physiological responses. Second, participants often enjoy watching film clips, thus enhancing their engagement. Third, as movie viewing is a familiar and enjoyable activity, it allows researchers to elicit strong negative emotions such as sadness or anger without crossing ethical lines. Therefore, viewing videos depicting authentic mathematical and pedagogical situations may increase the reliability of teachers' ER measurement.

(E) The subscales of the DESR and the ERQ will be correlated.

## Methods

### Sample size

Power analysis was conducted using G*power^78,79^, based on ƒ^2^ = 0.15, medium effect sizes, power > 80%, with a-priori alpha set at 0.05. The power analysis for the Pearson correlation showed 76 participants were needed. In addition, for multiple regression based on ƒ^2^ = 0.1, medium effect sizes, power > 80%, with a-priori alpha set at 0.05, 143 participants were required.

However, although the sample size for structural equations modeling (SEM) is a critical issue, there is no consensus in the literature on the appropriate sample size. N = 100–150 is usually considered the minimum^80^. Simulation studies show a reasonable sample size for a simple confirmatory factor analysis (CFA) model is about N = 150^81^. Our sample size for SEM was 165 (with missing data).

### Participants

The sample included 165 STEM teachers in middle and high schools (ages 17–64 years; M = 43.38 years, SD = 10.21; 69.1% females). Seniority of the teachers in the sample ranged from 1 to 35 years (M = 13.24 years, SD = 9.68). With respect to education, 24% of the teachers had a BA/BEd, 68% had an MA/MEd, 5% had a PhD, and 3% had other. Most taught in regular education (84%); some taught in special education classes in regular schools (13%), and a minority taught in special education schools (3%). The teachers' reports showed that 86% of them did not receive any training in ER. As for the main areas of knowledge taught, 41% reported that they only taught math, 41% said they only taught science, and the rest reported teaching more than one STEM subject. In the sample, 39% had converted to teaching from another field (e.g., high-tech, engineering).

### Procedure

Approval to conduct the study was received from the Human Research Ethics Committee at the administering institution (Approval No.: RB2109PD) and from the central scientist of the Ministry of Education (Approval No. 12471). An advertisement for participation in the study was distributed through social networks (Facebook, WhatsApp etc.). The ad mentioned participation in research on ER for STEM teachers. The teachers signed informed assent and consent prior to participation. Participant responses were recorded by Qualtrics Survey Platform, via an anonymous link sent to participants. The link included questionnaires that were randomly presented and three videos with changed order. All responses were anonymous and confidential. Data were compiled by Qualtrics and analyzed using IBM Statistical Package for Social Sciences, JASP, and SPSS. Exclusion criteria included subject of teaching (non-STEM teachers) and completion of only one questionnaire. Participants were compensated with a modest gift certificate. Overall, 165 STEM teachers met the criteria; 11 teachers did not meet the criteria, and their results were omitted. We confirm that all methods were carried out by relevant guidelines and regulations.

#### Ethical considerations and protection of participants’ rights

As in other psychological approaches to studies of stress management, a small risk is that reflecting on stress may evoke memories of highly traumatic stressful events in some individuals. Thus, the questionaries included information on the authority to be addressed in cases when difficult or traumatic thoughts arose^82^.

### Variables and measurement

#### Demography: background questionnaire

In the background questionnaire, teachers were asked demographic questions (age, gender, country of birth etc.). They were also asked questions about their education history and teaching training (e.g., Seniority- how many years have you been teaching?; Do you have training in special education?; Did you convert to teaching from another field?; What is your main teaching subject?.), the nature of the school where they taught, and the population they taught (e.g., average grade of students, number of students in the class, percentage of students estimated to have math anxiety, learning disabilities, attention disorders, lack of motivation to learn.). In addition, the teachers were asked if they had previously received training in ER.

#### ER

To evaluate the teachers' ER, we used two self-report questionnaires: Difficulties in Emotion Regulation Scale (DERS) and the well-known Emotion Regulation Questionnaire (ERQ). It is important to note that the DERS and the ERQ were developed based on different conceptualizations of ER^83^. While the ERQ draws on theory stemming from basic affective science with the aim of assessing individual differences in two particular ER strategies, reappraisal and suppression^48^, the DERS is a comprehensive measure of difficulties in ER, with the aim of assessing multidomain (i.e., cognitive, affective, behavioral) aspects of emotion dysregulation. This scale measures an integrative conceptualization of ER as involving not just the modulation of emotional arousal, but also the awareness, understanding, and acceptance of emotions, and the ability to act in desired ways, regardless of emotional state^84^.

##### Emotion regulation questionnaire (ERQ)

The ERQ^85^ was used to measure the teachers’ use of reappraisal and suppression strategies. The ERQ includes ten items; six measure reappraisal frequency (e.g., ‘I control my emotions by changing the way I think about the situation I'm in’), and four measure expressive suppression frequency (e.g., ‘I control my emotions by not expressing them’). Items are rated on a 7-point Likert-type scale from 1 (strongly disagree) to 7 (strongly agree). Higher scores on each scale indicate greater use of reappraisal or suppression strategies. The total score of the frequency of the use of each strategy is assessed by the average score of the relevant subscale. In three general community samples, the traditional 2-factor model (comprised of cognitive reappraisal and expressive suppression factors) was replicated and was an excellent fit to the data; cognitive reappraisal (α = 0.89–0.90) and expressive suppression (α = 0.76–0.80) scores had acceptable to excellent levels of internal consistency reliability^86^. In our study, the internal reliability of ERQ reappraisal was α = 0.86, and the internal reliability of ERQ suppression was α = 0.86.

##### Difficulties in emotion regulation questionnaire (DERS)

The DERS^87^ was used to measure multiple facets of ER. The questionnaire contains 36 items on a 5-point Likert scale ranging from 1 (almost never) to 5 (almost always) and includes six subscales: nonacceptance of emotional responses (items 11, 12, 21, 23, 25, 29); goal-directed behavior (items 13, 18, 20, 26, 33); impulse control difficulties (items 3, 14, 19, 24, 27, 32); lack of emotional awareness (items 2, 6, 8, 10, 17, 34); limited access to emotion regulation strategies (items 15, 16, 22, 28, 30, 31, 35, 36); and lack of emotional clarity (items 1, 4, 5, 7, 9). In addition, items 1, 2, 6, 7, 8, 10, 17, 20, 22, 24, and 34 are reverse-coded. Higher scores indicate greater difficulties in ER. The DERS has been found to have high internal consistency, good test–retest reliability, and adequate construct and predictive validity^87^. In our study, the internal reliability of the various factors was the following: nonacceptance of emotional responses α = 0.89; goal-directed behavior α = 0.84; impulse control difficulties α = 0.85; lack of emotional awareness α = 0.63 (without item 34); limited access to emotion regulation strategies α = 0.86; lack of emotional clarity α = 0.75 (without item 1). The total internal reliability of the DERS questionnaire was α = 0.94. ‘Difficulties in ER’ was a variable representing the summed score of the DERS (range 36–180)^84^. Higher scores indicated greater difficulties in ER. We also reported the six DERS subscales.

#### Teachers’ stress inventory (TSI)

The TSI was developed by Fimian^88^ and translated into Hebrew by Rudeina Badir, Bar Ilan University^89^. In our study, the teachers were asked to indicate the intensity of the sensations they experienced on a 5-point Likert scale ranging from 1 (not significant) to 5 (very significant), The higher the score, the higher the pressure experienced. The questionnaire includes 49 items and contains ten categories of factors affecting stress among teachers: time management, work-related pressures, professional stress, professional investment, discipline and motivation, emotional manifestations, fatigue manifestations, heart health-related manifestations, gastronomic manifestations, and behavioral manifestations. Fimian^88^ found the total internal reliability was high, α = 0.93; the internal reliability for each category was also high and ranged from α = 0.80 to α = 0.90. In our study, the internal reliability of the factors was the following: time management α = 0.77, work-related pressures α = 0.85, professional stress α = 0.81, professional investment α = 0.77, discipline and motivation α = 0.91, emotional manifestations α = 0.83, fatigue manifestations α = 0.85, heart health-related manifestations α = 0.81, and gastronomic manifestations α = 0.82. The total internal reliability of the TSI questionnaire was α = 0.95.

#### Well-being questionnaire

We used the Life Satisfaction Index (LSI) to measure the well-being of the teachers who participated in the study. The questionnaire was created by Wood et al*.*^90^ and translated into Hebrew by Shmotkin^91^. The questionnaire has five items on a Likert scale. Participants answer how much they agree with the item on a scale from 1 (very opposed) to 7 (very much agree). A sample item is: ‘I am satisfied with my life’. The total scores range from 5 to 35. Diener et al*.*^92^ reported high internal reliability (α = 0.83) and internal consistency (α = 0.82). In our study, the internal reliability was α = 0.88. Note that 82% of the sample completed the LSI.

#### Teachers' Burnout (TB) questionnaire

The TB^93^ questionnaire was used to measure burnout as a result of teachers’work at school. The questionnaire includes 14 items rated on a 6-point scale, with responses ranging from 1 (never) to 6 (always) describing teachers' feelings in three dimensions: burnout exhaustion (BE) (items 2, 12, 8, 6, 1), burnout lack of self-fulfillment (BL) (items 7, 4, 10, 9, 14), and burnout depersonalization of students (BD) (items 3, 5,1 1, 13). Teachers are asked to rate how often they have felt burnout in these areas in the last two to three months. The domains were generated by factor analysis using the Varimax method and the Oblimin method^93^. Internal reliability of the TB questionnaire according to Friedman^93^: α = 0.90. Internal reliability of the factors: exhaustion α = 0.90; lack of self-fulfillment α = 0.82; depersonalization α = 0.79. I. In our study, internal reliability of the factors: exhaustion α = 0.91; lack of self-fulfillment α = 0.86; depersonalization α = 0.85. Total score α = 0.92. Note that 82% of the sample completed the TB questionnaire.

#### Online Videos

Three short (1–2 min) online videos of a classroom situation were introduced in the link the teachers received. The videos were in the participants’ native language and were taken from the Simulation Center of a closed university website (https://halev-biu.org.il/videos/), with the permission of the Center but also with a request not to distribute the videos due to copyright.

In the first video, during math class, one student successfully solves an exercise on the board, but a girl in the class does not participate. The teacher overhears a conversation she has with the boy next to her. She says she doesn't understand anything, and she wants to drop to a lower level, even though it is only a month before the final exam. The girl crumples the page and puts her head on the table in despair. In the second video, the teacher meets personally with a student who failed a math test to give her feedback on the mistakes she made. The student complains and tells the teacher how desperate she is. In the third video, the teacher tries to teach a heterogeneous class, a class where there are gaps in the level of understanding of the different students.

The teachers were asked to watch the three videos. They were required to respond to seven questions after they watched each video, and the average across the three videos was calculated for each of three variables: video emotion (four questions), video suppression (one question), and video reappraisal (two questions). The questions asked teachers to rate their feelings and thoughts on a 7-point Likert-type scale from 1 (strongly disagree) to 7 (strongly agree). We determined the average response for each variable. Video emotion referred to the intensity of the negative emotions felt by the teachers while watching the videos. Two questions referred to the intensity of the emotion of anger the video might evoke, and two referred to the intensity of the emotion of sadness the video might evoke. This was averaged across the three videos. The internal reliability of this variable was α = 0.86. Video suppression referred to the average use of suppression in the three videos (e.g., ‘In a similar situation in class, I make sure not to express the negative emotion that the situation evokes in me’). The internal reliability of this variable was α = 0.78. Two questions related to dealing with emotions through a strategy of reappraisal (e.g., ‘In a similar situation in class, I try to find the advantages and focus on positive aspects’). The internal reliability of this variable was α = 0.87.

Note that 92% of the sample watched the videos and answered the accompanying questionnaire.

## Results

### Stress, well-being, burnout, and ER in teachers

We used Pearson correlations to test for relations between study variables. As shown in Table 1, as hypothesized, there were significant correlations between well-being, burnout, and stress among participating STEM teachers. The higher the well-being, the lower the burnout and stress. Also as hypothesized, there were significant correlations between difficulties in ER and well-being, burnout, stress, reappraisal, and suppression. One exception was the DERS emotional awareness subscale which was not significantly correlated with burnout and stress. Overall, the greater the difficulties in ER, the lower the well-being and use of a reappraisal strategy, and the higher the burnout, stress, and use of a suppression strategy.

**Table 1:** Means, standard deviations, and correlations between study variables.

Variables		n	M (SD)	1	2	3	4	5	6	7	8	9	10	11	12
Well-being	1	136	4.91 (1.12)												
Burnout	2	135	3.20 (0.93)	− 0.532***											
stress	3	165	2.79 (0.63)	− 0.456***	0.661***										
Difficulties in ER	4	165	70.52 (19.04)	− 0.394***	0.353***	0.349***									
Nonacceptance	5	165	12.91 (5.17)	− 0.282**	0.258**	0.299***	0.775***								
Goal	6	165	12.00 (4.14)	− 0.243**	0.291**	0.307***	0.738***	0.454***							
Impulse	7	165	11.28 (4.56)	− 0.295***	0.278**	0.209**	0.855***	0.528***	0.590***						
Awareness	8	165	11.11 (2.77)	− 0.251**	0.069	− 0.035	0.470***	0.260**	0.161*	0.349***					
Strategies	9	165	16.25 (5.63)	− 0.418***	0.376***	0.406***	0.881***	0.640***	0.616***	0.690***	0.251**				
Clarity	10	165	6.97 (2.58)	− 0.308***	0.293**	0.262**	0.706***	0.375***	0.370***	0.658***	0.451***	0.560***			
Reappraisal	11	165	5.18 (1.01)	0.160	− 0.059	− 0.063	− 0.227**	− 0.122	− 0.177*	− 0.180*	− 0.087	− 0.274***	− 0.136		
Suppression	12	165	3.50 (1.46)	− 0.165	0.101	0.092	0.251**	0.219**	0.024	0.151	0.220**	0.288***	0.247**	0.091	
Seniority	13	165	13.24 (9.68)	0.147	− 0.143	− 0.126	− 0.052	0.035	− 0.038	− 0.076	0.089	− 0.086	− 0.170*	0.092	− 0.091

*p < 0.05; **p < 0.01; ***p < 0.001.

Note: 82% of the sample completed the well-being and burnout questionnaires.

Well-being: sum of the items; burnout: average score; Stress: average score; difficulties in ER: summed score of the DERS (range 36–180); Nonacceptance, Goal, Impulse, Awareness, Strategies, and Clarity: six subscales of the DERS; Reappraisal and Suppression: mean score of each subscale in the ERQ; Seniority: in years.

Our third hypothesis was disproved; seniority (measured in years of teaching) was not correlated with any of the variables, suggesting seniority does not play a significant role in predicting ER skills, stress, well-being, or burnout among STEM teachers.

### Responses to authentic mathematical and pedagogical situations

We examined the correlations between the two ER self-report questionnaires (ERQ and DERS) and participants’ responses to three authentic mathematical and pedagogical situations shown in videos. In line with our fourth hypothesis, there was a significant positive correlation between video emotion (the intensity of the negative emotions, anger/sadness, the teacher felt when watching the videos), the use of the suppression strategy reported in the ERQ, and difficulties in ER reported in the DERS, both in the general measure of the DERS and for five of the six DERS subscales. One subscale, lack of emotional awareness, did not have a significant correlation but it was positive. In addition, there was a positive correlation between video reappraisal and the use of a reappraisal strategy reported in the ERQ. The more the teachers reported using reappraisal in the ERQ, the more they used reappraisal in the authentic situations shown in the videos (Table 2).

**Table 2:** Means, standard deviations, and correlations between videos and ER questionnaires.

Variables		n	M	SD	1	2	3
Video Emotion	1	152	3.21	1.01			
Video Suppression	2	152	4.65	1.61	0.118		
Video Reappraisal	3	152	4.81	1.28	0.081	0.465***	
Difficulties in ER	4	165	70.52	19.04	0.312***	0.013	− 0.127
Nonacceptance	5	165	12.91	5.17	0.310***	0.092	− 0.107
Goal	6	165	12.00	4.14	0.205*	0.012	− 0.072
Impulse	7	165	11.28	4.56	0.282***	− 0.019	− 0.098
Awareness	8	165	11.11	2.77	0.011	− 0.079	− 0.002
Strategies	9	165	16.25	5.63	0.306***	0.012	− 0.164*
Clarity	10	165	6.97	2.58	0.217**	− 0.019	− 0.089
Reappraisal	11	165	5.18	1.01	− 0.005	0.033	0.277***
Suppression	12	165	3.50	1.46	0.229**	0.019	− 0.046

*p < 0.05; **p < 0.01; ***p < 0.001.

Note: 92% of the sample watched the videos and answered the accompanying questionnaire.

Video emotion: average score of the intensity of the emotions of anger and sadness felt by the teachers while watching the three videos; Video suppression: average use of suppression in the three videos; Video reappraisal: average use of reappraisal in the three videos; Difficulties in ER: summed score of the DERS (range 36–180); Nonacceptance, Goal, Impulse, Awareness, Strategies, and Clarity: six DERS subscales; Reappraisal and Suppression: mean score of each subscale in the ERQ; Seniority: in years.

### Difficulties in ER Skills

Table 3 shows the correlations between the subscales of the DESR and the ERQ. As predicted by the fifth hypothesis, all the correlations between ERQ reappraisal and the DERS were negative. The results suggest the two questionnaires are related but distinct, indicating they assess different aspects of ER.

**Table 3:** Subscales of DESR and ERQ: means, standard deviations, and correlations.

Subscales of DESR	ERQ Reappraisal	ERQ Suppression
1. Nonacceptance of emotional responses	− 0.122	0.219**
2. Goal− directed behavior	− 0.177*	0.024
3. Impulse control difficulties	− 0.180*	0.151
4. Lack of emotional awareness	− 0.087	0.220**
5. Limited access to ER strategies	− 0.274**	0.288**
6. Lack of emotional clarity	− 0.136	0.247**

*p < 0.05; **p < 0.01; ***p < 0.001.

### ER difficulties and stress, well-being, and burnout: are they related?

We used a structural equation model (SEM) with a maximum-likelihood estimation (MLS) to test whether stress, difficulties in ER, and well-being predicted burnout; whether stress and difficulties in ER predicted well-being; and whether stress predicted difficulties in ER. The model generated a good fit: χ^2^(71) = 105.52, *p* = 0.005, χ^2^/*df* = 1.49, CFI = 0.969, TLI = 0.961, RMSEA [90% CI] = 0.054 [0.031, 0.075], SRMR = 0.051. All factor loadings of the indicator variables for each latent variable were significant at *p* < 0.001.

The model we used did not include the ERQ measures. We should note that a similar model with the ERQ measures had a good model fit (χ^2^(94) = 186.23, *p* < 0.001, χ^2^/*df* = 1.98, CFI = 0.922, TLI = 0.901, RMSEA [90% CI] = 0.077 [0.061, 0.093], SRMR = 0.067), but the ERQ measures were not significant (*p* > 0.05) . Moreover, and more importantly, there was a significant difference (χ^2^(23) = 89.53, *p* < 0.001) between the models, supporting the presented model over a model with the ERQ measures.

**Figure 1:**
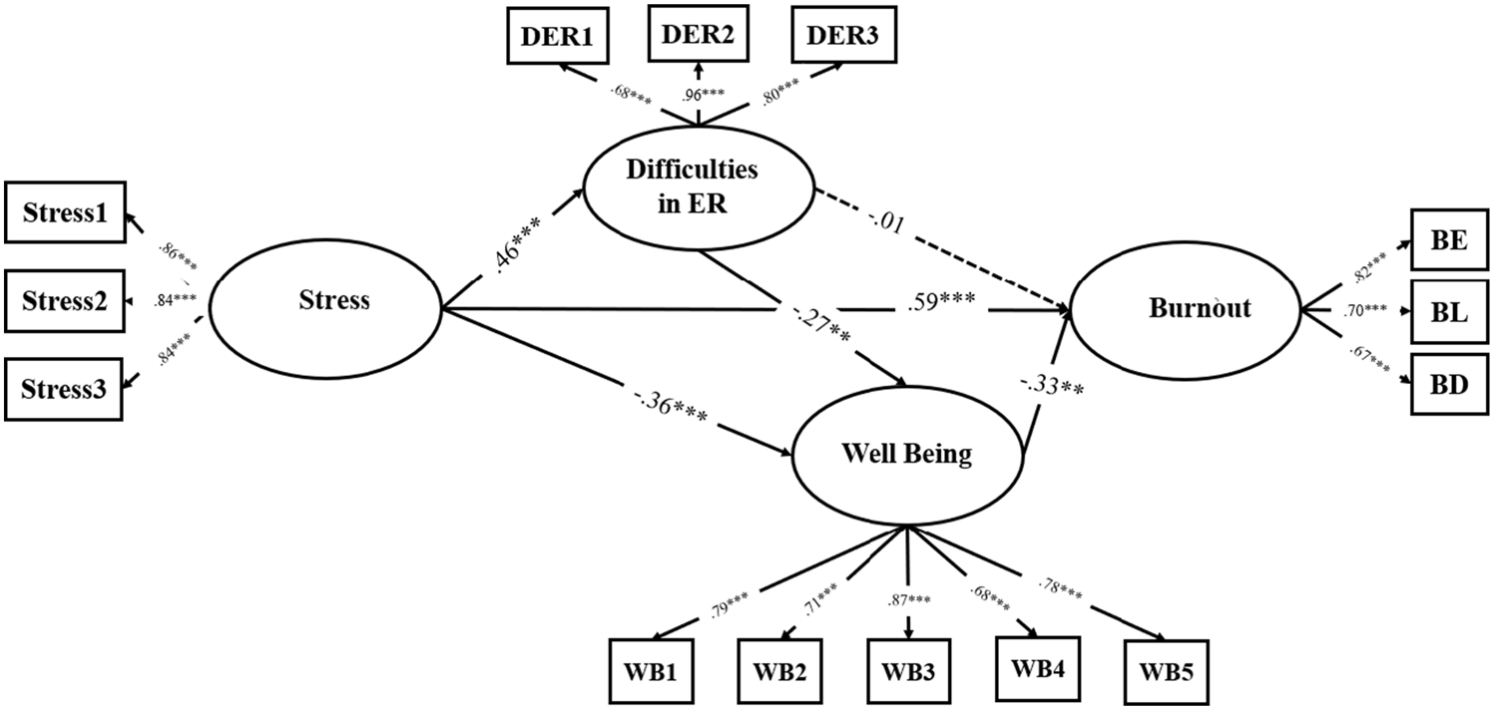
SEM Model. *p < 0.05; **p < 0.01; ***p < 0.001. Note: Stress1: Parceling of index 1, 4, and 7 in the stress questionnaire. Stress2: Parceling of index 2, 5, and 8 in the stress questionnaire. Stress3: Parceling of index 3, 6, and 9 in the stress questionnaire. DER1: Parceling of the factors of nonacceptance and awareness in the DERs. DER2: Parceling of the factors of Strategies and Clarity in the DERs. DER3: parceling of the factors of goal and impulse in the DERs. BE: burnout exhaustion. BL: burnout lack. BD: burnout depression. WB1 to WB5: the five indexes in the well-being questionnaire.

The results are presented in Fig. 1. Most of the path coefficients were significant in the analysis. Stress predicted well-being negatively and difficulties in ER and burnout positively. Difficulties in ER predicted well-being negatively. In addition, well-being predicted burnout negatively. Moreover, the coefficient effect of stress predicting well-being through the mediation of difficulties in ER was significant. The coefficient effect of stress predicting burnout through the mediation of well-being was also significant. But the coefficient effect of stress predicting burnout through the mediation of difficulties in ER was not significant.

Bootstrapping coefficients based on 5000 resamples and the lower and upper bounds of 95% confidence intervals are presented in Table 4. Most of the effects in the structural equation model were significant. The bootstrapping confidence intervals’ lower and upper bounds of the direct effects from stress to well-being, difficulties in ER, and burnout, the direct effect from difficulties in ER to well-being, the direct effect from well-being to burnout, the indirect effect from stress to well-being through difficulties in ER, and the indirect effect from stress to burnout through well-being were not zero. Therefore, teachers' stress had an effect on well-being through the mediation of difficulties in ER, and teachers' stress had an effect on burnout through the mediation of well-being. Note that D2 in the model refers to two difficulties in ER: limited access to ER strategies and lack of emotional clarity.

**Table 4:** Direct and indirect effects and 95% confidence intervals for the model.

Model path	Coefficient	Percentile 95% CI	
		Lower	Upper
Direct effect			
Teacher Stress → Burnout	0.59*	0.27	0.82
DER → Burnout	− 0.01	− 0.18	0.21
WB → Burnout	− 0.33*	− 0.58	− 0.09
Teacher Stress → WB	− 0.36*	− 0.55	− 0.20
DER → WB	− 0.27*	− 0.46	− 0.07
Teacher Stress → DER	0.46*	0.31	0.60
Indirect effect			
Teacher Stress → DER → Burnout	− 0.01	− 0.09	0.10
Teacher Stress → WB → Burnout	0.12*	0.04	0.25
Teacher Stress → DER → WB	− 0.13*	− 0.23	− 0.03

*p < 0.05.

## Discussion

The impact of a teacher's emotional state on the classroom environment is well documented in the literature^94^. Teachers who experience enjoyment while teaching have more positive attitudes when students struggle, spend more time on teaching, and exhibit effective pedagogical behaviors^95^. Meanwhile, stress can negatively impact teacher-student relationships, teacher behavior, and classroom quality^96,97^.

Teachers regulate their emotions every day in school and use various ER strategies in classroom situations. Suppression is the most common strategy reported by teachers to control their emotional expression and hide their negative emotions in response to students' behavioral problems^67^. The use of suppression may be helpful to manage the classroom^29^, but frequent use consumes cognitive resources^85^ and can lead to difficulty connecting emotionally with students, a lack of social support, and increased stress and burnout^49,58,65–67^. Regulating negative emotions through an adaptive strategy such as reappraisal leads to more positive interactions and a supportive response to student emotions^29,50^. Teachers who use an adaptive strategy to regulate emotion, such as reappraisal, have better teaching styles (autonomy support and structure motivation) and better classroom management skills^47,58,59^.

Supporting our first two hypotheses, ER skills predicted well-being, stress, and burnout among the STEM teachers in our sample. The greater the difficulties in ER, the lower the well-being and use of reappraisal, and the higher the burnout, stress, and use of suppression. However, unexpectedly and not in line with previous studies, seniority was not correlated with any of the variables, and the third hypothesis was not supported. In other words, it cannot be assumed that the ER of teachers is solely dependent on their seniority measured as years of teaching, and apparently ER does not improve over time. In support of our fourth hypothesis, we found a correlation between the self-reported responses in the questionnaires and the genuine reactions when viewing videos depicting authentic mathematical and pedagogical situations, a finding which supports teachers’ reports on their strategies for regulating emotions, and presents evidence of the use of video as another effective assessment tool for ER^75–77^.

A major finding was that STEM teachers' stress influenced their well-being through the mediation of difficulties in ER. As we hypothesized, increased levels of stress^21,59,66^ were linked to emotion dysregulation. Emotion dysregulation, in turn, was associated with well-being and burnout^58,59,66^. It is well-established that stress can have a negative impact on a teacher's well-being^98^. When a teacher's well-being is high, the risk of burnout is reduced.

The SEM analysis of the DERS questionnaire identified two factors as more significant in mediating the relationship between stress and well-being: limited access to ER strategies and lack of emotional clarity (DERS2 in the SEM model). These findings suggest targeted interventions can be developed to address ER difficulties in teachers.

The results also suggest the evaluation of ER should go beyond examining only basic individual differences in the use of reappraisal and suppression as evaluated in the ERQ questionnaire^48^. In our opinion, and in line with our results, we suggest the evaluation of ER should include a deeper and broader evaluation of difficulties in ER and assess multidomain (i.e., cognitive, affective, behavioral) aspects of emotion dysregulation, according to the assessment in the subscales of the DERS^84^. This is supported by recent research that found only modest associations between the DERS-36 and the ERQ subscales of reappraisal and suppression. This is not surprising because they have different theoretical foundations^83^.

Our study has potential implications for intervention programs for teachers and pre- service teachers^99^. The results indicate the need to find practical ways to promote the reappraisal of stress arousal in pedagogical programs and to study the contribution of intervention programs to teachers' and students' educational and emotional outcomes. A stress reappraisal intervention should be designed to assist teachers in managing the stress associated with their work. Stress reappraisal interventions have value for several reasons. First, they are grounded in theory, with a strong evidence base demonstrating proof‐of‐concept^100,101^. Second, they have translatability from the laboratory to multiple contexts^102^. Third, they are non-invasive and require limited time and resources from participants^100,101^.

A practical way to improve ER is through the practice of mindfulness meditation^103–107^. Mindfulness interventions have been shown to improve mindfulness skills and decrease psychological distress^108–110^. In educational settings, practicing mindfulness helps to reduce stress^109^ and contributes to overall well-being^110^. Recent research found that reappraisal has a greater effect on performance than either informational control or mindfulness interventions^111^, but research on how teachers regulate their emotions and which strategies are effective in the classroom is sparse^29^, with a dearth of research-based interventions.

Teachers’ emotions and their ability to regulate them are arguably as important as the more widely studied education or general experience measures. The ability to efficiently regulate emotions should be considered a crucial component in teaching^29^, especially in STEM topics. All teachers face challenges in classroom teaching. Strengthening their emotional skills, resources, and support sources will reduce inhibitory factors and barriers and promote successful integration of students who need unique responses and reduce teacher dropouts from the education system. Given the current teacher shortage crisis, particularly in STEM fields^3,112^, it is important to address the stress faced by teachers.

The findings reveal connections between the teachers’ reports on regulating emotions in the questionnaires and their reports on the intensity of the negative emotions experienced watching the videos. It is possible that teachers and pre-service teachers could practice ER with simulation^113,114^, a common practice in medical and nursing studies^115^. There are several simulation centers in teacher education institutions in Israel, and they can be a place for teachers and pre-service teachers to practice using ER tools.

Admittedly, the study had some limitations. First, the sample was relatively small, and this may affect the generalizability of the findings. Second, the interpretation of the study's results should be approached with caution because of the diverse nature of STEM education systems worldwide. Future studies should examine a larger sample of teachers, including comparative studies across countries. Third, the study focused on teachers' ER using teacher self-report questionnaires. Future research that incorporates the perspectives of both teachers and their students may offer a more complete understanding.

To conclude, our main argument is that ER skills are a crucial component of teaching^8,29,50^, especially in STEM fields^4^. While recent studies show stress influences a teacher's well-being^98^, we found ER plays a key role in mediating the relationship between stress and well-being, and well-being, in turn, reduces burnout. Therefore, our research suggests the need to provide tools for teachers to regulate their emotions in the classroom, and by doing so, to improve their well-being. This would have advantages for both teachers and students^52^ and might reduce the number of teachers leaving the profession, especially STEM teachers.
